# The Clock Drawing Test: A Valid Screening Instrument for Dementia Detection in Low-Educated Patients?

**DOI:** 10.3390/geriatrics10060164

**Published:** 2025-12-12

**Authors:** Janique Boots-van der Heiden, Jos van Campen, Tessa Kooistra, Irene van de Vorst, Miriam Goudsmit

**Affiliations:** 1 Department of Geriatrics, OLVG Hospital, 1061 AE Amsterdam, The Netherlands; 2 Department of Palliative Care, Isala Hospital, 8025 AB Zwolle, The Netherlands; 3 Department of Geriatrics, Maasstad Hospital, 3079 DZ Rotterdam, The Netherlands; 4 Department of Geriatrics, Alrijne Hospital, 2353 GA Leiderdorp, The Netherlands; 5 Independent Researcher, 1061 AE Amsterdam, The Netherlands; 6 Department of Psychiatry & Medical Psychology, OLVG Hospital, 1061 AE Amsterdam, The Netherlands

**Keywords:** dementia, education, age, clock drawing test, screening instrument, neuropsychological test

## Abstract

**Objective:** The non-verbal nature of the Clock Drawing Test (CDT) suggests it is a suitable cognitive screening instrument for populations with lower educational levels and/or language barriers. This study evaluates whether the CDT is a valid screening instrument for low-educated patients and includes a qualitative analysis of CDT errors. **Method:** A total of 503 participants were included, divided into four groups (dementia, MCI, no cognitive impairment, and other diagnosis), based on a clinical diagnosis by a geriatrician. Educational levels were categorized into four groups: no education and low, middle, and high education. CDT scores were assessed using the seven-point scoring system (Freedman), and two cutoff points were evaluated. **Results:** Results showed that in all education categories, the dementia group scored significantly lower on the CDT compared to the non-dementia group. The difference was smallest in participants with no education. Two cut-off points were assessed: <4 and <3. A cut-off of <4 showed better sensitivity versus <3, particularly for low-educated groups. A cut-off of <3 provided better specificity versus <4. Error analysis showed that errors made by low-educated participants without dementia were similar to those of patients with dementia. **Conclusions:** These findings show that the CDT (both total score and qualitative error analysis) has limited value in dementia case-finding in low-educated groups. The CDT is recommended primarily for middle- and high-educated groups.

## 1. Background

The prevalence of dementia is increasing rapidly, and physicians will increasingly be confronted with dementia amongst elderly. Today, a more diverse population, including both migrants and non-migrants, with a wider range of educational backgrounds, visits general practitioners and memory clinics for cognitive assessment. Not only does the minority ethnic population have a higher risk of developing dementia, diagnosing dementia in these groups is often complex due to language and cultural barriers, low education, or illiteracy [1,2,3,4]. As a result, the diagnosis of dementia in these groups is often delayed or not made at all, and treatment and advice may be postponed or lacking [2,3]. Limited education or illiteracy has a significant impact on numerous neuropsychological tests within a wide range of cognitive domains [5,6]. Standard cognitive screening tests may thus not be suitable, and there is need for effective methods to accurately screen for cognitive dysfunction among populations with limited education [5,6].

A widely used cognitive screening instrument is the Clock Drawing Test (CDT). The CDT is considered to be a good screening test for dementia and cognitive dysfunction [7,8,9]. It assesses multiple cognitive domains, such as executive functions, visuospatial organization, visuoconstructional skills, psychomotor coordination, and working memory. It serves as a valuable complement to the Mini-Mental State Examination (MMSE), particularly by addressing the MMSE’s limited assessment of executive functions [7,9]. The CDT has practical advantages for clinical use because it is easy to administer, is not threatening to the patient, and takes a short amount of time to complete [9]. Moreover, the sensitivity and specificity of the CDT for diagnosing dementia is relatively high but depends on which scoring system is used. Over 20 quantitative scoring systems and subsequent scales have been developed. The scale developed by Shulman is most often used in clinical practice, but other scales are as accurate in detecting dementia [10,11].

The CDT is administered verbally, with no written instructions, no verbal output, and written language required. Therefore, it has been suggested that the CDT is less sensitive to influences such as culture, language, and education [7,10]. However, recent research in diverse populations suggests that the CDT might not be suitable for lower-educated and/or illiterate individuals [5,10,12,13,14]. For example, visuoconstructional difficulties in illiterate subjects can easily be misinterpreted as signs of cognitive dysfunction [5,12]. Similarly, effects of age and education on CDT performance have been demonstrated across cultures [15,16].

Whereas the quantitative validity of the CDT has received considerable attention in research, so far, only a few studies have examined the types of errors made on the CDT and their associations with education, culture, and language. A Korean study conducted such an error analysis and found that healthy, elderly participants with a limited educational background made similar errors to those made by patients with mild dementia of the Alzheimer’s type [17].

This study aims to investigate the validity of the CDT as a screening instrument for dementia in low-educated patients. The second goal is to determine the best sensitivity/specificity combination (i.e., cut-off scores) for discriminating between dementia and no-dementia. Furthermore, we also performed a systematic qualitative analysis to determine common error types in the group with low education compared to patients with dementia.

## 2. Materials and Methods

### 2.1. Data Source and Setting

**Figure 1:**
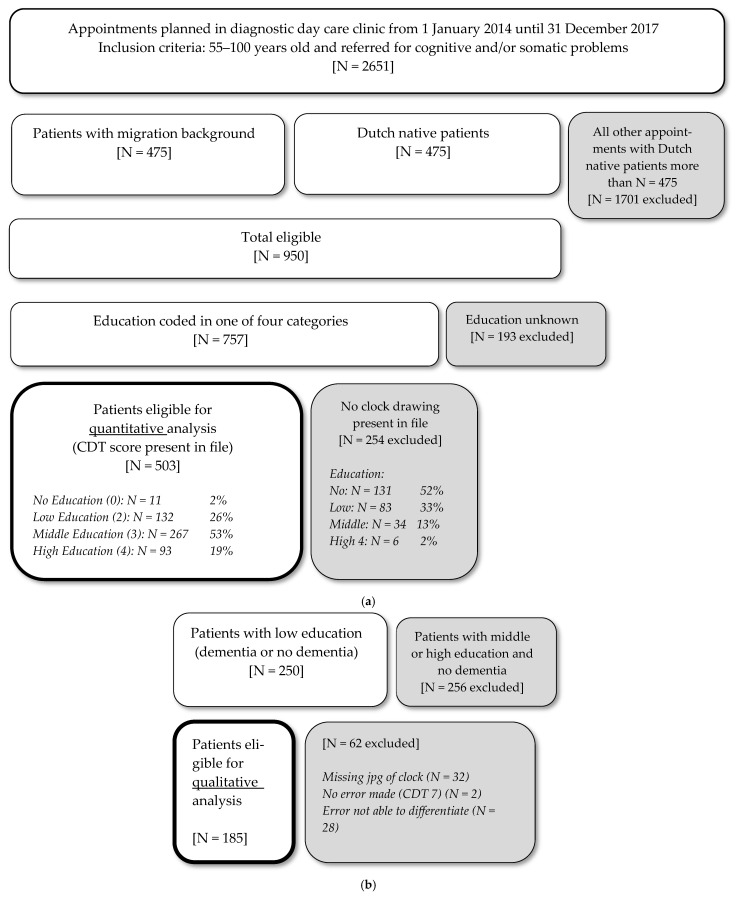
Flow chart of inclusion and exclusion of patients: (**a**) quantitative analysis; (**b**) qualitative analysis.

This study analyzed the data obtained from the database of the PROPS study (PROgnosis and Presenting Symptoms in ethnically diverse patients with dementia), which is a retrospective cohort study containing data from 950 patients. Included in the PROPS study were patients aged 55 to 100 years with cognitive and/or somatic problems, who had been referred to the diagnostic geriatric day clinic of the Slotervaart hospital in Amsterdam, the Netherlands, between 1 January 2014 and 31 December 2017. Approval of the local Medical Ethical committee of OLVG was obtained [nr. WO 19.208], and the research was completed in accordance with Helsinki Declaration. Informed consent was waived because of the retrospective nature of the study and because the analysis used anonymous chart reviews. Patients who were unable to complete cognitive tests—for instance, due to paralysis, aphasia, refusal to cooperate, or serious vision or hearing disabilities—were excluded. For the present study, all patients who completed the CDT and whose education level was known were included (Figure 1). As shown in the flow diagram, only 11 of 142 patients without education were able to draw a clock in the first place, which made the “no education” group very small compared to the other educational subgroups.

### 2.2. Cohorts

Subjects were divided into four groups based on their clinical diagnosis: no cognitive impairment, Mild Cognitive Impairment (MCI), dementia, and other diagnoses (such as delirium, Parkinson’s disease, alcohol abuse, schizophrenia, and depression). Dementia was diagnosed according to the National Institute on Aging Alzheimer’s Association (NIA-AAA) criteria [18], and MCI was diagnosed according to the core clinical criteria of the NIA-AAA workgroup [19]. For our main analysis, we contrasted the patients with dementia with the patients with no cognitive impairment or MCI, leaving the other diagnosis group out. To classify the level of education, we used the Dutch Verhage scale [20]. This scale contains seven categories, and for the purpose of this study, we constructed the following subgroups: no education, elementary school (low education), secondary education (middle education), and tertiary education (high education).

For the qualitative analyses, we specifically strived to compare those without dementia and low or no education to those with dementia. Patients without dementia with middle or high education were excluded from qualitative analysis.

### 2.3. Instruments

All participants underwent a standardized diagnostic evaluation conducted by a geriatrician. This included a clinical interview with both the patient and the caregiver, a physical examination, neuropsychological screening assessment performed by trained nurses, and brain imaging on indication. For participants from minority backgrounds with a language barrier, a professional interpreter was present during the visit. Cognitive assessments included the Mini-Mental State Examination (MMSE), the Seven Minute Screen (7 MS), including the Clock Drawing Test (CDT), and the Informant Questionnaire on Cognitive Decline in the Elderly (IQCODE) [21,22,23]. For illiterate patients, the MMSE score was corrected for missing items using the following formula: total score * (30/number of points obtained) [24]. There is no consensus in the literature regarding the validity of the different quantitative scoring systems for the CDT [7]. We used the 7-point scoring system as used in the 7 min screen based on the scoring system developed by Freedman [23,25]. The scoring system evaluates specific features of a drawn clock such as number placement, spacing, and hand positioning. Scores range from 0, representing the lowest performance, to 7, representing the highest performance.

Qualitative error analyses of the CDT were performed using the modified qualitative error analysis of Rouleau [26]. The patterns of the analyzed errors were as follows: graphic difficulties, stimulus-bound responses, conceptual deficits, spatial and/or planning deficit, and perseveration. All the clocks drawn by patients with dementia and without dementia in the no education and low education subgroups were analyzed. Of some patients, the CDT number score was present, but the clock drawing itself was not added to the file; thus, it could not be qualitatively scored. Interrater agreement with this scoring system is generally high (>90%) [27]. The researchers that determined the qualitative clock scores were both geriatricians in training with experience in scoring cognitive screening instruments. To assess interrater agreement, a subset of in total 25 CDTs were evaluated by 2 clinical geriatricians in training (TK and BS). Disagreement was found to be less than 10% (those were solved through careful scoring and deliberation among testers). The remaining CDTs were analyzed by a single researcher (TK).

### 2.4. Data Analysis

Statistical analyses were performed using SPSS version 22. Continuous variables were expressed as means ± standard deviations or medians with interquartile ranges, depending on data distribution. Categorical variables were expressed as frequencies and percentages. Differences between groups (no cognitive impairment, MCI, and dementia) were tested using chi-square tests or Kruskal–Wallis tests, as appropriate. Subgroup analyses were performed to assess differences between education levels and CDT scores using chi-square tests. The level of statistical significance for all tests was set at *p* < 0.05. Sensitivity, specificity, Youden’s index, positive and negative predictive values, and positive and negative likelihood ratios were calculated for different cut-off points.

## 3. Results

### 3.1. Baseline Characteristics

A total of 503 patients were included in the analysis, of whom 130 had no cognitive impairment, 171 were diagnosed with MCI, 180 with dementia, and 22 with another diagnosis (e.g., delirium, Parkinson’s disease, alcohol abuse, schizophrenia, or depression). In Table 1, we illustrate which groups were used in which analysis.

**Table 1:** Classification of subgroups included in each analysis.

Quantitative Analyses					
	Clinical Diagnosis				
	No Cognitive Impairment	MCI ^1^	Dementia	Other Diagnosis ^2^	Total
Table 2	N = 130	N = 171	N = 180	N = 22	503
Figure 2 Boxplot CDT score per education group	N = 301		N = 180	N = 22 not included	481
Table 3 Sensitivity specificity	N = 301		N = 180	N = 22 not included	481
Table 4 Logistic regression	N = 481			N = 22 not included	481
**Qualitative Analyses**					
No and low education	56		55	N = 22 not included	185
Middle or High education	N = 256 not included		74	N = 22 not included	

Note: ^1^ Mild Cognitive Impairment. ^2^ Other diagnoses included delirium, Parkinson’s disease, alcohol abuse, schizophrenia, and depression.

**Table 2:** Demographic characteristics, Clock Drawing Test results and cut-off scores (N = 503).

	Clinical Diagnosis					
	No Cognitive Impairment	MCI ^5^	Dementia	Other Diagnosis	Total	*p*
N	130	171	180	22	503	
Gender, male (N, %)	53 (41%)	64 (37%)	79 (44%)	9 (41%)	205 (41%)	X^2^ (3) = 1.51; *p* = 0.68
Age (M, SD)	77 (8.3)	80 (8.0)	82 (7.3)	78 (9.0)	80 (8.1)	K-W ^6^ (3) = 26.3; *p* ≤ 0.001
Education						
No education (N, %)	5 (4%)	2 (1%)	4 (2%)	0 (0%)	11 (2%)	X^2^ (9) = 28.0; *p* ≤ 0.001
Low education ^1^ (N, %)	29 (22%)	31 (38%)	69 (14%)	3 (14%)	132 (26%)	
Middle education ^2^ (N, %)	69 (53%)	103 (60%)	83 (46%)	12 (55%)	267 (53%)	
High education ^3^ (N, %)	27 (21%)	35 (20%)	24 (13%)	7 (32%)	93 (18%)	
Ethnicity						
Dutch (N, %)	85 (66%)	129 (75%)	128 (72%)	16 (76%)	358 (72%)	X^2^ (12) = 9.9; *p* = 0.62
Turkish (N, %)	8 (6%)	3 (2%)	8 (4%)	0 (0%)	19 (4%)	
Moroccan (N, %)	5 (4%)	5 (3%)	5 (3%)	0 (0%)	15 (3%)	
Surinamese/Hindustani (N, %)	2 (%)	0 (0%)	2 (1%)	0 (0%)	4 (1%)	
Other (N, %)	29 (22%)	34 (20%)	35 (20%)	5 (24%)	103 (21%)	
MMSE ^4^ (M, SD)	26 (3.8)	24 (3.5)	19 (5.6)	23 (5.8)	23 (5.3)	K-W ^6^ (3) = 128; *p* ≤ 0.001
**CDT score ** **^7^ (M, SD)**	5.1 (1.41)	4.3 (1.71)	3.2 (1.94)	3.7 (1.88)	4.1 (1.88)	
**Cut-off < ** **3 (N; %) ** **^8^**	9 (7%)	26 (15%)	69 (38%)	7 (32%)	111 (22%)	X^2^ (3) = 51.0; *p* ≤ 0.001
**Cut-off < ** **4 (N; %) ** **^9^**	22 (17%)	56 (33%)	105 (58%)	11 (50%)	194 (39%)	X^2^ (3) = 59.0; *p* ≤ 0.001

^1^ Elementary school, Verhage 1–2. ^2^ Secondary education, Verhage 3–5. ^3^ Tertiary education, Verhage 6–7. ^4^ Mini Mental State Examination (0–30), corrected, if necessary, for missed items due to illiteracy (total score ∗ (30/number of points obtained)). ^5^ Mild cognitive impairment. ^6^ Kruskal–Wallis test. ^7^ Clock Drawing Test score according to Freedman (0–7), with 0 representing the worst score and 7 representing the best score. ^8^ CDT score below cut-off 3, indicative of cognitive impairment. ^9^ CDT score below cut-off 4, indicative of cognitive impairment.

**Figure 2:**
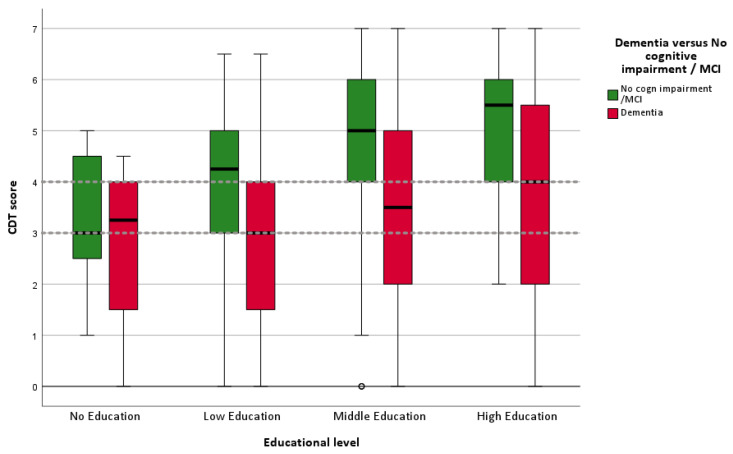
Boxplot of CDT scores for four education categories (N = 481). Dotted lines represent cutoff score of 3 and 4. Hollow circle represents an outlier.

**Table 3:** Sensitivity, specificity, Youden index, and likelihood ratios for the Clock Drawing Test, contrasting dementia with no cognitive impairment/MCI for CDT cutoff < 3 and cutoff < 4 (N = 481).

	N	Sensitivity% (95% CI ^1^)	Specificity% (95% CI)	Youden Index ^2^	PPV ^3^ (95% CI)	NPV ^4^ (95% CI)	LR+ ^5^ (95% CI)	LR− ^6^ (95% CI)
**Cutoff < 3**								
Total	481	38% (31–46%)	88% (84–92%)	0.26	66% (58–74%)	71% (68–73%)	3.30 (2.29–4.74)	0.70 (0.62–0.79)
No education	11	25% (0.63–81%)	71% (29–96%)	−0.04	33% (6–80%)	63% (44–78%)	0.88 (0.11–6.88)	1.05 (0.50–2.19)
Low education	129	46% (34–59%)	87% (75–94%)	0.33	80% (67–89%)	58% (52–64%)	3.48 (1.74–6.96)	0.62 (0.49–0.79)
Middle education	255	34% (24–45%)	88% (82–92%)	0.22	57% (45–69%)	73% (70–76%)	2.76 (1.67–4.56)	0.75 (0.64–0.89)
High education	86	33% (16–55%)	94% (84–98%)	0.27	67% (40–86%)	78% (73–83%)	5.17 (1.71–15.58)	0.71 (0.53–0.95)
**Cutoff < 4**								
Total	481	58% (51–66%)	74% (69–79%)	0.32	57% (52–63%)	75% (71–78%)	2.25 (1.79–2.83)	0.56 (0.47–0.68)
No education	11	75% (19–99%)	43% (10–82%)	0.18	43% (24–64%)	75% (31–95%)	1.31 (0.56–3.09)	0.58 (0.09–3.90)
Low education	129	74% (62–84%)	62% (48–74%)	0.36	69% (61–76%)	67% (57–76%)	1.93 (1.36–2.74)	0.42 (0.27–0.66)
Middle education	255	51% (39–62%)	77% (70–83%)	0.28	52% (43–60%)	76% (72–80%)	2.23 (1.58–3.16)	0.64 (0.51–0.81)
High education	86	38% (19–59%)	81% (69–90%)	0.19	42% (27–61%)	77% (71–82%)	1.94 (0.94–4.00)	0.78 (0.56–1.08)

Note: ^1^ CI, confidence interval; ^2^ Youden index = (sensitivity + specificity) − 1; ^3^ PPV, positive predictive value; ^4^ NPV, negative predictive value; ^5^ LR+, positive likelihood ratio; ^6^ LR−, negative likelihood ratio.

**Table 4:** Logistic regression analysis with CDT score (the dependent variable) < 3 and <4.

Variable	B ^1^	SE B ^2^	Wald Test	*p*	OR ^3^ (95% CI ^4^)
CDT score (the dependent variable) < 3					
Age	0.050	0.015	10.649	0.001	1.051 (1.020–1.083)
Gender	0.083	0.234	0.127	0.722	1.087 (0.687–1.721)
Ethnicity	0.065	0.057	1.309	0.252	1.067 (0.955–1.192)
Educational level	−0.493	0.162	9.288	0.002	0.611 (0.445–0.839)
Constant	−4.602	1.298	12.570	<0.001	0.010
CDT score (the dependent variable) < 4					
Age	0.041	0.013	9.821	0.002	1.041 (1.015–1.068)
Gender	−0.168	0.205	0.670	0.413	0.846 (0.566–1.263)
Ethnicity	0.112	0.050	5.069	0.024	1.119 (1.015–1.234)
Educational level	−0.752	0.144	27.194	<0.001	0.472 (0.356–0.626)
Constant	−2.559	1.090	5.507	0.019	

Note: ^1^ B, estimated unstandardized regression coefficients; ^2^ SE B, standard error of B; ^3^ OR, odds ratio; ^4^ 95% CI, 95% confidence interval of odds ratio.

Small but significant group differences were observed in age, with patients with dementia being older on average. Educational levels also varied across diagnostic groups. Because the group with no formal education was very small (N = 11), it was combined with the low-education group for qualitative analyses. As expected, MMSE scores differed significantly among the four groups, ranging from a mean score of 19 in the dementia group to 26 in the group without cognitive impairment (see Table 2).

### 3.2. Clock Drawing Test Performance for Patients with and Without Dementia per Educational Level

In Table 2, the mean CDT scores for all diagnostic groups are presented. Patients with dementia scored lower on the CDT (M = 5.1, SD = 1.41) than those with MCI (M = 4.3, SD = 1.71) and those without dementia (M = 3.2, SD = 1.9; *p* ≤ 0.001). In Figure 2, the CDT performances of patients with dementia versus no dementia (no cognitive impairment + MCI) are shown via a boxplot, stratified into four educational levels. The boxplot gives a graphical summary of the CDT scores, including the median, quartiles, and outliers. It is clearly visible that a substantial proportion of patients without dementia and low or no education score below the cutoffs of <3 or <4.

In Table 3, the sensitivity, specificity, Youden index, predictive values, and likelihood ratios of the CDT scores are listed per educational level for two different cut-off points (<3 and <4). The best cut-off, based on the Youden Index in the groups with no or low education, would be <4.

In Table 4, the results of a logistic regression analysis are presented, with CDT score (<3 and <4) being the dependent variable and demographic variables, namely, age, gender, ethnicity, and educational level, being the predictors. For both cutoff points, age and educational level are significant predictors.

### 3.3. Error Analysis

**Figure 3:**
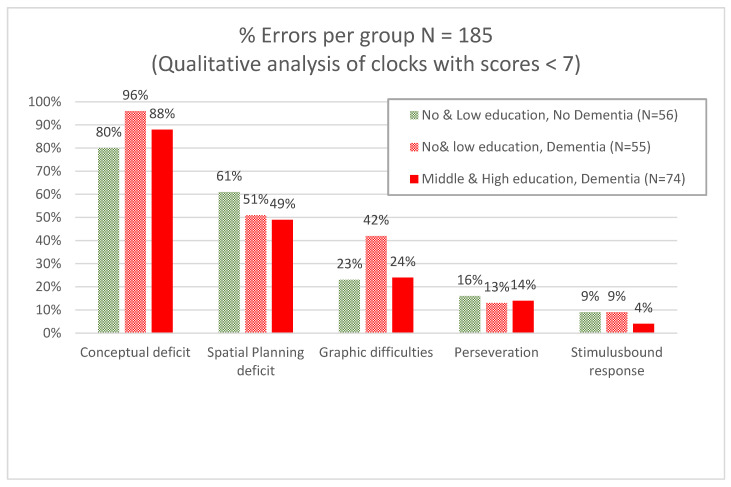
Error analysis for contrasting dementia with no cognitive impairment/MCI stratified per level of education (N = 185).

A total of 218 clocks were analyzed for error analysis, of which 30 clocks were without errors, or else the errors could not be differentiated into one of the subcategories. Therefore, a total of 185 clocks with errors were considered, of which 129 were drawn by patients with dementia (55 with no and low education; 74 with middle and high education) and 56 were drawn by patients without dementia but with low or no education. In Figure 3, percentage of error types are given per patient group. The most common error in all groups was a conceptual deficit, and the second most common error was a spatial/planning deficit. Examples of clock drawings by several participants are provided in the Supplementary Material.

## 4. Discussion

### 4.1. Summary of Findings and Comparison to Literature

The aim of this study was to investigate whether the Clock Drawing Test (CDT) is a valid screening instrument for dementia detection in low-educated patients. In addition, a qualitative study of CDT errors was conducted.

To examine the clinical validity of the CDT, we compared the CDT scores of patients with a clinical diagnosis of dementia to those without dementia, stratified by educational level. Our findings show a positive association between educational level and CDT score. As illustrated in Figure 2, patients with dementia consistently scored lower on the CDT than those without dementia within the same education group. The difference in CDT scores between the dementia and no-dementia groups widened with increasing educational level and was smallest among patients with no formal education. Interestingly, patients with dementia and a high educational level still outperformed non-demented patients with a low educational level on the CDT. This indicates a limitation for the clinical use of the CDT for individuals with low educational backgrounds. De Noronha et al. [28] concluded that the CDT is suitable for assessing mainly visuoconstructional praxis and providing an overall impression of cognitive function among individuals, independently of years of education, but performance is significantly lower among illiterate individuals, indicating that literacy has a substantial influence on test outcomes. In two studies involving an elderly Chinese population, CDT performance was found to be significantly influenced by both age and education [15], as well as by years of education [29].

In clinical practice, different cut-off scores for the CDT are used. In this study, we compared the sensitivity and specificity of cut-off scores of <4 to <3 in the different educational groups. Sensitivity is higher with a cut-off score of <4 than <3, especially for the two lower educational groups (sensitivity of 75% in the group with no education and sensitivity of 74% in the group with low education). Specificity is higher with a cut-off score of <3 than with <4 in all educational groups. A cut-off of <3 could be more useful to exclude or rule-out dementia in patients with a low clinical suspicion on dementia diagnosis. In groups with a higher suspicion of dementia, a cut-of score of <4 could lead to yet higher suspicion, especially for higher-educated groups. Postulating a base rate of 30% dementia in a memory clinic population, a CDT score of <4 would give a post-test probability of 67.5% (LR+ of 2.25) (Table 3). Leung et al. [29] provided different cut-off scores based on the Chinese CDT scoring criteria for various educational levels to differentiate early-stage Alzheimer’s dementia patients from age-matched healthy controls and found that the optimal cut-off points were higher for individuals with minimal education. Ainslie and Murden [30] found that the specificity decreased with lower education and concluded that the CDT is a poor single screening test for dementia in low-educated patients.

We performed a qualitative error analysis in a subgroup of our sample (N = 185) to examine if patients with and without dementia could be determined based on error type [31]. We did not find differences in error types for patients with and without dementia; the most common errors in all groups were “conceptual deficits”. This was also found in the smaller study by Kim and Chey concerning 28 patients with mild dementia of the Alzheimer’s type and 28 cognitively healthy volunteers [17].

In everyday practice, cognitive screening of individuals with suspected dementia will almost always encompass more screening instruments than CDT alone. We advise combining CDT with broader cognitive screening tests such as the Rowland Universal Dementia Assessment Scale (RUDAS) [31], which is more suitable for a lower-educated group than the traditionally favoured Mini Mental State Examination (MMSE) [32]. Also, adding information from another source, such as an informant interview such as the Informant Questionnaire for Cognitive decline of the Elderly (IQCODE), enhances predictive value [33].

### 4.2. Strengths and Limitations

This study is a retrospective study of the data of the PROPS study (PROgnosis and Presenting Symptoms in ethnically diverse patients with dementia). To classify the level of education, we used the Dutch Verhage scale, which is based on the number of years of education. However, educational level does not take into account the quality of education, which differs between countries and regions. Also, it can be postulated that exposure to clocks and importance of clock reading differ for patients coming from “western” countries compared to patients of “non-western” origin. Staios et al. [34] examined the validity of visuoconstructional tests in a sample of older Greek–Australian immigrants compared to matched sample of patients with Alzheimer’s disease. They found that cognitively healthy immigrants with low education appear to be at a disadvantage when completing visuoconstructional drawing tests, and that their performance may be misinterpreted as indicating cognitive impairment. In the present study, all patients with no education and 36% of low-educated patients had a migration background. Unfortunately, due to the small number of non-educated and illiterate patients in our group, it was not feasible to calculate the influence of migration background independently of education.

The dementia diagnosis in our study partly relied on the CDT score (among many other parameters, such as an interview and other cognitive screening tests), leading to possible risk of incorporation bias as the test is included in the reference standard. This can cause an overestimation of sensitivity and specificity. Because of the retrospective nature of this study, incorporation bias could not be fully avoided. Importantly, because of this bias, the results are likely inflated; thus, the CDT’s value as a dementia screening tool may be even poorer than presented. Furthermore, the absence of independent clinical verification represents an additional limitation as the diagnostic process relied on assessments within the same clinical setting.

In the main comparisons of our study, the no-dementia group also included patients diagnosed with mild cognitive impairment. Additionally, no distinction was made regarding the type of dementia. Potential differences between dementia subtypes may therefore have influenced CDT performance, which could not be accounted for in the present analysis. It will be useful to explore the severity—and various subtypes—of dementia in future research.

In our study, a limited number of patients without formal education and/or who were illiterate were included. Out of the 756 patients for whom the educational level was known, 232 had a missing CDT, and of these patients, 74% had no or low education. It is postulated that these patients have a missing CDT because of their inability to draw a clock and/or their embarrassment about having their drawing evaluated. Consequently, this indicates that the outcomes of our study might have been even stronger if these patients had been included, and it also supports our conclusion that the CDT is not a suitable test for patients with limited education.

To summarize, compared to the aforementioned studies, we included a large cohort of patients with diverse backgrounds and employed a naturalistic study design. Additionally, the qualitative addition of our study is superior to those which have been conducted to date.

### 4.3. Implications for Research and Practice

Future studies should include larger cohorts of individuals who are illiterate or low-educated and explore the underlying reasons why some patients do not complete the CDT in order to obtain more conclusive evidence. One potential alternative to the CDT is the Papadum Test [35], which may be more suitable for individuals with limited literacy or formal education. Additionally, younger generations are increasingly accustomed to digital clocks; hence, their ability to read and draw traditional analog clocks is declining. Further validation studies are needed to evaluate these alternatives.

## 5. Conclusions

These findings demonstrate that the CDT is a less useful cognitive screening instrument for patients with no or low formal education. In these populations, CDT results should be interpreted with caution. When dementia is clinically suspected and the patient is able to complete the CDT, we recommend using a cut-off score of 3, especially in patients with low or no education. When taking a closer look at the types of errors being made in the CDT, there is no difference between participants with dementia and participants without dementia but with low education; therefore, the CDT cannot be used to differentiate between these groups.

Therefore, we advise using the CDT primarily for individuals with moderate to high levels of education.

## Data Availability

The data presented in this study are available on request from the corresponding author due to privacy reasons.

## Supplementary Materials

The following supporting information can be downloaded at https://www.mdpi.com/article/10.3390/geriatrics10060164/s1, Figure S1: Examples of Clocks by participants of the study.
