# Supplementary material for: The Clock Drawing Test: A Valid Screening Instrument for Dementia Detection in Low-Educated Patients?
Source: Geriatrics (Basel). 2025 Dec 12;10(6):164. doi: 10.3390/geriatrics10060164 (PMC12732869; doi:10.3390/geriatrics10060164)
Supplement: Supplementary file 1 [file geriatrics-10-00164-s001.zip › geriatrics-3928033-supplementary.pdf]

Supplementary material. Examples of Clocks by participants of the study

Details of each participant provided below

A	B	C	D	E
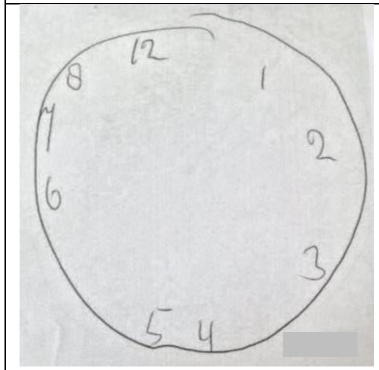	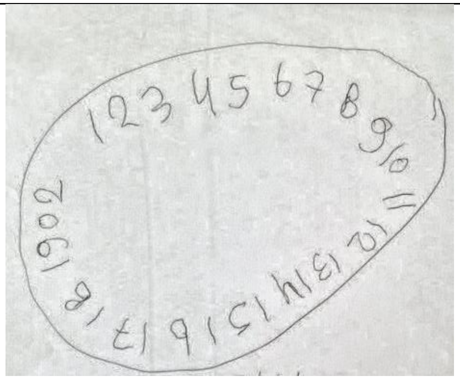	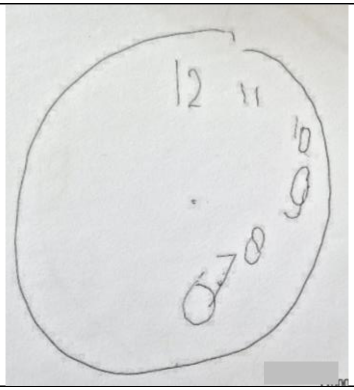	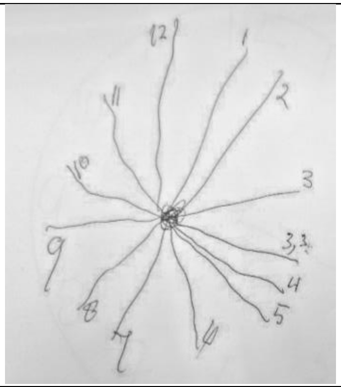	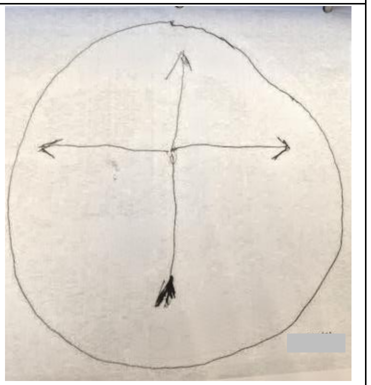
Woman, 86 years old, education only primary school, no dementia	Woman, 74 years old, no education, no dementia	Man, 75 years old, education high, Lewy Body Dementia	Woman, 94 years old, education high (university), Dementia of the Alzheimers type	Man, 80 years old, education high, dementia of the Alzheimer's type
